# Deciphering the Pharmacological Mechanisms of the Huayu-Qiangshen-Tongbi Formula Through Integrating Network Pharmacology and *In Vitro* Pharmacological Investigation

**DOI:** 10.3389/fphar.2019.01065

**Published:** 2019-09-23

**Authors:** Zihao Wang, Ke-Gang Linghu, Yuanjia Hu, Huali Zuo, Hao Yi, Shi-Hang Xiong, Jinjian Lu, Ging Chan, Hua Yu, Run-Yue Huang

**Affiliations:** ^1^State Key Laboratory of Quality Research in Chinese Medicine, Institute of Chinese Medical Sciences, University of Macau, Macau, China; ^2^Department of Rheumatology and Immunology, The Second Affiliated Hospital of Guangzhou University of Chinese Medicine, Guangdong Provincial Hospital of Chinese Medicine, Guangzhou, China; ^3^HKBU Shenzhen Research Center, Shenzhen, China; ^4^School of Chinese Medicine, Hong Kong Baptist University, Hong Kong, Hong Kong; ^5^Guangdong Provincial Key Laboratory of Clinical Research on Traditional Chinese Medicine Syndrome, Guangzhou, China

**Keywords:** rheumatoid arthritis, network pharmacology, traditional Chinese medicine, Huayu-Qiangshen-Tongbi formula, *in vitro* validation

## Abstract

Rheumatoid arthritis is a chronic inflammatory autoimmune disease, causing articular and extra-articular dysfunctions among patients, and it could result in irreversible joint damages or disability if untreated. A traditional Chinese medicine formula, Huayu-Qiangshen-Tongbi (HT) formula, has been observed successful in controlling rheumatoid arthritis progression in traditional Chinese medicine clinics. In this study, we conducted a systematic analysis of the HT formula with a purpose of proposing for its potential mechanism of action using network pharmacological methods. The potential targets of the formula were collected and screened according to the topological features of their protein–protein interaction network, and we subsequently validated our prediction results through *in vitro* experiments. We proposed that the HT formula could interfere with the bone metabolism and the inflammatory pathways of the body. The experimental validation results indicated that HT formula could exhibit anti-inflammatory effects by regulating several signaling pathways specifically the Toll-like receptor signaling pathway, phosphoinositide-3-kinase–Akt signaling pathway, hypoxia-inducible factor 1 signaling pathway, mitogen-activated protein kinase signaling pathway and activator protein 1 signaling pathway.

## Introduction

Rheumatoid arthritis (RA) has been regarded as one of the most common autoimmune inflammatory diseases, and effective treatments for RA require the use of disease-modifying antirheumatic drugs (DMARDs). Although DMARDs have been frequently prescribed in clinic, patients often suffered from unwanted side effects from the treatment, such as the myelosuppression and the injuries of liver and kidney (Obiri et al., 2014). Traditional Chinese medicine (TCM) herbal formula has gained attention as an alternative remedy practiced for over a thousand years due to their observed efficacy with reduced side effects (Venkatesha et al., 2011; He et al., 2014).

Huayu-Qiangshen-Tongbi (HT) formula, a TCM herbal formula for treating RA, was developed by the Department of Rheumatology and Immunology from the Guangdong Provincial Hospital of Chinese Medicine, and its clinical efficacy was based on more than 20 years of clinical observation and clinical practice. The composition of the formula reflects the academic thoughts regarding the RA treatment by Liang-Chun Zhu, the well-known TCM Master in Rheumatology, and Liang Liu. The formula is composed of these 10 herbs: *Salvia miltiorrhiza* Bge. (Danshen), *Dioscorea nipponica* Makino (Chuanshanlong), *Astragalus mongholicus* Bge. (Huangqi), *Paeonia lactiflora* Pall. (Baishao), *Saussurea involucrata* (Kar. & Kir.) Sch.Bip. (Xuelian), *Eucommia ulmoides* Oliv. (Duzhong), *Drynaria roosii* Nakaike (Gusuibu), *Dipsacus inermis* Wall. (Chuanxuduan), *Rehmannia glutinosa* (Gaertn.) DC. (Dihuang), and *Glycyrrhiza uralensis* Fisch. (Gancao). Lu et al. observed the treatment for a total of 77 patients with RA, and they concluded that a combination of HT formula with methotrexate (MTX) could achieve an equivalent efficacy to that of leflunomide with MTX after 12 weeks of treatment, and the patients went through a combination therapy of using HT formula and MTX experienced less adverse effect than that of the other treatment (Lu et al., 2019). Although the HT formula is commonly used in clinic in China, there is a lack of pharmacological researches to elucidate the mechanisms of action (MOA) on the HT formula treating RA largely due to the complex composition of the formula, limiting further development of the prescription.

In order to overcome the technical barriers for deciphering the MOA for TCM prescriptions, recently, network pharmacological methods were developed by integrating dry and wet lab techniques. In this way, the individual chemical ingredients contained in the herbal formula could be analyzed for their therapeutic effects without jeopardizing the completeness of the treatment (Li and Zhang, 2013). Through systematically studying the information revealed in a network, the network analysis method could provide the prediction results regarding the MOA of the herbal formulae. In addition, the preliminary results from the network analysis could also offer a direction for the following pharmacological verification, facilitating the holistic examinations of the TCM formulae. Inspired by this promising method, various researchers in the field of rheumatology had applied network pharmacological methods to study and explore the anti-RA effects of the TCM formulae. Zhang et al. proposed the potential rationale behind the interactions among the herbs in the Wu Tou Tang decoction (WTD) when treating RA. They predicted the potential MOA of the WTD by analyzing the RA protein–protein interaction (PPI) network and identified the candidate effector molecules of the WTD (Zhang et al., 2013). Li et al. collected and analyzed 871 anti-RA herbal prescriptions from a clinic using an herb–compound–target–disease coherent network, and they proposed the synergistic effects underlying the core herbs of these prescriptions as well as the pharmacological mechanisms underlying these prescriptions combating RA (Li et al., 2015). By integrating network analysis, *in vivo* as well as *in vitro* experiments, Guo et al. concluded that WTD could act on RA progression by inhibiting inflammatory responses through modulating the C–C chemokine receptor type 5 signaling pathway in macrophages (Guo et al., 2017).

**Figure 1:**
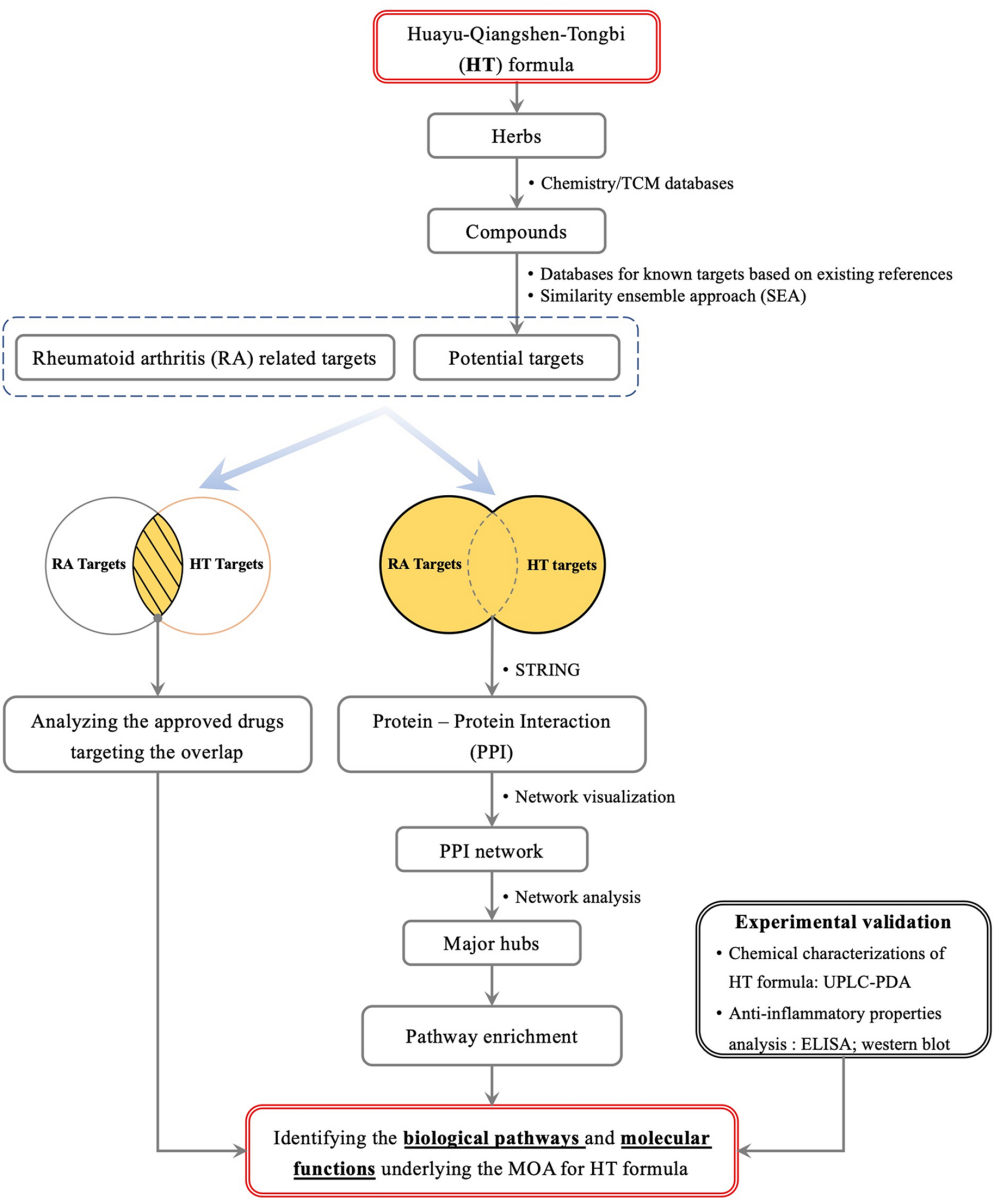
A scheme illustrating the general workflow of studying the HT formula using a systematic method.

In this study, we aim to explore active compounds and action mechanisms of the HT formula by combining computational predictions based on network pharmacology, chemical analysis, and *in vitro* investigations. As illustrated in **Figure 1**, we collected and analyzed the potential targets of the HT formula for a systematic study of the formula. The obtained targets were compared to the RA-related targets for deducing the potential clinical indications of the HT formula in treating RA. In order to study the RA-related interactions involved by the human body after consuming the HT formula, the PPI data among the potential targets of the HT formula and the RA-related targets were visualized in a network, and the potential MOA was proposed by studying the major hubs of the PPI network. Finally, the *in vitro* experiments were integrated to validate the anti-inflammatory properties of the HT formula. This work may provide a valuable reference to the further quality control, product development, as well as clinical applications of the HT formula.

## Material and Methods

### Network Pharmacology Analysis of the HT Formula

#### Data Preparation

The chemical information of the herbs contained in the HT formula was manually curated from an integration of three databases, including the Shanghai Institute of Organic Chemistry of Chinese Academy of Sciences, Chemistry Database (last updated on June 17th, 2016) (Shanghai Institute of Organic Chemistry), TCM Database @ Taiwan (last updated on March 25th, 2014) (Chen, 2011), as well as SciFinder database. The existent compound–target interaction (CTI) information for the HT formula was acquired from the current literatures using ChEMBL database (version 23) (Gaulton et al., 2017), and only the human targets were retained for further studying.

The phytochemicals obtained were submitted to the Similarity Ensemble Approach (Keiser et al., 2007) for predicting potential CTIs, and the human targets with high confidence (Tc ≥ 0.57) were selected from the prediction results as the potential targets of the phytochemicals.

The RA-related targets were collected from DrugBank database (version 5.0) (Wishart et al., 2018), Online Mendelian Inheritance in Man (last updated in May, 2018) (Amberger and Hamosh, 2017), the Genetic Association Database (last updated in August, 2014) (Becker et al., 2004), and Kyoto Encyclopedia of Genes and Genomes (KEGG) pathway database (last updated in May, 2018) (Kanehisa et al., 2017).

The PPI data for the acquired targets were obtained from the STRING database (V10.5) (Szklarczyk et al., 2016). PPIs with medium confidence based on the confident scores defined by the STRING developers were retained for network analysis.

#### Network Construction and Analysis

The PPIs of the HT targets in addition to the RA-related targets were visualized using Cytoscape (version 3.6.1) (Shannon et al., 2003). A hub in the HT targets–RA targets PPI network was defined as the node having a degree of more than twofold of the median degree of all the nodes (Li et al., 2007). The major hubs in the PPI network of the hubs were identified for the sake of screening the important hubs, and the major hubs were calculated based on four topological features, that is “Degree,” “Node betweenness,” “Closeness,” and “K-core value” (Zhang et al., 2017).

Pathway enrichment analysis for the major hubs was performed using the data from KEGG in the Database for Annotation, Visualization and Integrated Discovery (V6.8) (Huang et al., 2008; Huang et al., 2009).

### Experimental Investigations of the HT Formula

#### Herbal Extraction

The herbs were soaked in 600 ml of deionized water (five times water of the total weight of TCM) for 30 min. Then, the mixture was heated to boiling before being decocted for 30 min. The filtrate of the first decoction was collected, while the remaining herbs were treated with another 500 ml of deionized water for the second decoction at the boiling point for 60 min. The filtrate of the second decoction was combined with the filtrate from the first decoction and was centrifuged at 2,500 rpm for 10 min. The supernatant was subsequently concentrated using rotary evaporator, and the concentrate was frozen at −80°C for 24 h before acquiring its lyophilized powder.

#### Chemical Analysis

Danshensu sodium salt, 3-caffeoylquinic acid, caffeic acid, paeoniflorin, rutin, quercetin, rosmarinic acid, salvigenin, calycosin, and glycyrrhizic acid (the purities of all standards were higher than 98% by high-performance liquid chromatography analysis) were purchased from Chengdu Pufeide Biotech Co., Ltd. (Chengdu, China). Acetonitrile and methanol as high-performance liquid chromatography grade were purchased from RCI Labscan Limited (Thailand). Phosphoric acid as analytical grade was purchased from Sigma Chemicals Ltd. (St. Louis, MO, USA). Milli-Q water was prepared using a Milli-Q system (Millipore, MA, USA).

The quantifications of 10 compounds in the HT extract were performed by a Waters ACQUITY- Ultraperformance Liquid Chromatography (UPLC) Class system (Waters Corp., Milford, USA) coupled with an ACQUITY UPLC HSS T3 column (150 mm × 2.1 mm, 1.8 µm) maintained at 40°C. Elution was performed with a mobile phase of A (0.2% phosphoric acid in water) and B (0.2% phosphoric acid in acetonitrile) under a gradient program: 0–2 min, 2% B; 2–4 min, 2–8% B; 4–12 min, 8–12% B; 12–14 min, 12–20% B; 14–18 min, 20–25% B; 18–25 min, 25–40% B; 25–30 min, 40–60% B. The flowrate was 0.4 ml/min, and the injection volume was 2 μl. The analytes were monitored at the UV wavelength of 215 nm. Between the two injections, the column was washed with 100% B for 2 min and equilibrated with the initial mobile phase for 5 min.

#### Anti-Inflammatory Properties

RAW264.7 cells were obtained from the American Type Culture Collection (ATCC; Manassas, VA, USA). Cells were cultured in Dulbecco’s modified Eagle medium supplemented with 10% heat-inactivated fetal bovine serum and 1% P/S in an incubator with 95% humidity and 5% CO_2_ at 37°C. Cells were subcultured after scraping away from the flask (25 cm^2^; Thermo Fisher Scientific, MA, USA) when the cells reached 80% confluence. The cells were cotreated with the extracts in 96-well plates at the indicated concentrations for 24 h, and then, the cell viability was detected by 3-(4,5-dimethylthiazol-2-yl)-2,5-diphenyltetrazolium bromide (MTT) assay. The cells were pretreated with the extracts at the concentrations of 100, 200, and 400 μg/ml in 24-well plates for 1 h before they were stimulated with lipopolysaccharides (LPS, 1 μg/ml) for 24 h, and the supernatants were collected to determine NO release using Griess reagent, and the cytokines secretion was determined using ELISA kits (Neobioscience Technology Co., Ltd., Shenzhen, China). In addition, the cells were pretreated with the extracts at the concentrations of 100, 200, 400 μg/ml in six-well plates for 1 h first, and then, they were stimulated with LPS (1 μg/ml) for 1 or 12 h, respectively. The total proteins were extracted from the harvested cells using radioimmunoprecipitation assay buffer containing 1 mM phenylmethanesulfonyl fluoride and protease inhibitor cocktail. Relevant proteins from the network analysis were detected by western blotting analysis as previously described (Sang et al., 2018). Immunofluorescence staining was used to detect the expression of inducible nitric oxide synthase (iNOS) and the translocation of nuclear factor kappa B (NF-κB) p65 (Li et al., 2018).

### Statistical Analysis

Each experiment was performed in triplicate and was repeated for at least thrice. The Student’s *t* test was used to compare the two groups. ANOVA followed by Dunnett’s test were used to compare three or more groups. *P* < 0.05 was considered having statistically significant difference.

## Results

### Computational Predictions

#### Putative Targets and the Major Hubs of the HT Formula

A total of 800 unique compounds were identified from the 10 herbs of the HT formula. Using the data from ChEMBL (Gaulton et al., 2017), we collected 560 targets for the compounds in the HT formula. The experimentally validated CTI information and the predicted CTIs using the Similarity Ensemble Approach were combined to give a total of 739 unique targets responsible for the compounds contained in the HT formula.

Based on the databases selected, 287 human targets were collected as being related to the pathological mechanism of RA (**Table S1**), and 35 of them were found to be potentially targeted by the chemical constituents of the HT formula. In order to deduce the possible MOA from clinically prescribed drugs that were sharing the same targets with the HT formula, we compared the targets of the HT formula to the targets of the currently approved drugs recorded in the DrugBank database (Wishart et al., 2018) for RA treatment, and a total of 23 drugs were detected having overlapping targets with the HT formula (**Table 1**), including four kinds of DMARDs. These approved drugs have been frequently prescribed to RA patients due to their anti-inflammatory and analgesic effects (Wishart et al., 2018), indicating that the HT formula may function through a similar MOA when treating RA.

**Table 1:** List of the approved disease-modifying antirheumatic drugs (DMARDs) and nonsteroidal anti-inflammatory drugs (NSAIDs) sharing common targets with the Huayu-Qiangshen-Tongbi (HT) formula.

Approved Drugs	DrugBank ID	Shared targets	Type
Ibuprofen	DB01050	25	NSAID
Indomethacin	DB00328	21	NSAID
Diclofenac	DB00586	20	NSAID
Acetylsalicylic acid	DB00945	15	NSAID
Celecoxib	DB00482	10	NSAID
Flurbiprofen	DB00712	10	NSAID
Ketoprofen	DB01009	10	NSAID
Sulindac	DB00605	10	NSAID
Etodolac	DB00749	9	NSAID
Naproxen	DB00788	9	NSAID
Sulfasalazine	DB00795	9	DMARD
Meloxicam	DB00814	7	NSAID
Piroxicam	DB00554	7	NSAID
Diflunisal	DB00861	6	NSAID
Tolmetin	DB00500	6	NSAID
Fenoprofen	DB00573	5	NSAID
Leflunomide	DB01097	4	DMARD
Meclofenamic acid	DB00939	4	NSAID
Nabumetone	DB00461	4	NSAID
Tenoxicam	DB00469	4	NSAID
Azathioprine	DB00993	3	DMARD
Oxaprozin	DB00991	3	NSAID
Hydroxychloroquine	DB01611	2	DMARD

The interactions among the targets of the HT formula and the RA targets were determined using PPI data. Using the PPI data among the HT and RA targets, a PPI network for the targets of the HT formula and the RA targets was constructed. Since a node could be identified as a hub if its degree was more than twofold of the median degree of all the nodes in a network (Li et al., 2007), 150 hubs were identified in the HT–RA PPI network. To screen out the nodes playing critical roles in the network, we determined the major hubs of the hubs interaction network by calculating four topological indicators, including “Degree,” “Node betweenness,” “Closeness,” and “K-core value” (Zhang et al., 2017). The median values of the four indicators were 42.5, 0.00397, 0.634, and 34, respectively. The hubs showing values higher than the medians of the four indicators were determined as the major hubs of the network. Among the 41 major hubs identified (**Table S2**), 29 of them were the potential targets of the HT formula.

#### Potential Pathways Involved and Possible MOA for the HT Formula

**Figure 2:**
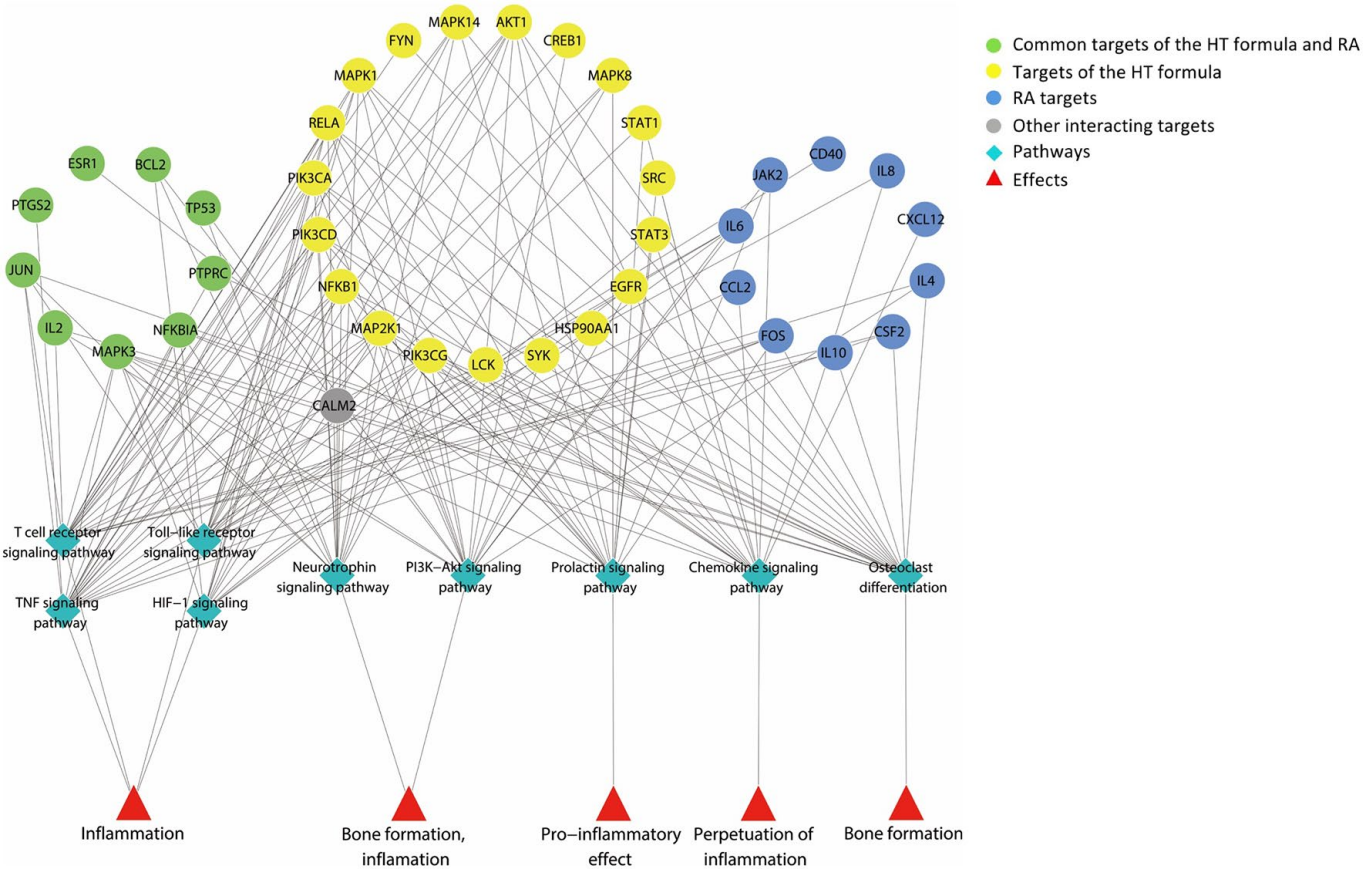
A graph illustrating the major hubs of Huayu-Qiangshen-Tongbi–rheumatoid arthritis (HT–RA) protein–protein interaction (PPI) network and their frequently associated pathways. The pathway enrichment analysis showed that the major hubs were often involved with the nine pathways shown in the graph, and these pathways participated in the bone metabolism and inflammation of RA.

The major hubs identified were submitted to the Database for Annotation, Visualization and Integrated Discovery (Huang et al., 2008; Huang et al., 2009) for pathway enrichment analysis based on the data from KEGG pathway database. As shown in **Figure 2**, the major hubs were mainly associated with the function of bone metabolism and the inflammatory pathways (**Table 2**).

**Table 2:** The major hubs of the Huayu-Qiangshen-Tongbi–rheumatoid arthritis (HT–RA) protein–protein interaction (PPI) network and their frequently associated pathways.

Term	Nodes	Gene IDs
Osteoclast differentiation	20	LCK, PTPRC, NFKBIA, PIK3CG, MAP2K1, MAPK3, NFKB1, IL2, JUN, IL4, PIK3CD, CSF2, PIK3CA, IL10, RELA, MAPK1, FYN, MAPK14, FOS, AKT1
T cell receptor signaling pathway	20	LCK, PTPRC, NFKBIA, PIK3CG, MAP2K1, MAPK3, NFKB1, IL2, JUN, IL4, PIK3CD, CSF2, PIK3CA, IL10, RELA, MAPK1, FYN, MAPK14, FOS, AKT1
TNF signaling pathway	19	CCL2, NFKBIA, PTGS2, PIK3CG, MAP2K1, MAPK3, NFKB1, IL6, CREB1, JUN, PIK3CD, CSF2, PIK3CA, RELA, MAPK1, MAPK14, FOS, AKT1, MAPK8
Prolactin signaling pathway	17	STAT1, SRC, PIK3CG, MAP2K1, MAPK3, NFKB1, ESR1, PIK3CD, JAK2, PIK3CA, RELA, MAPK1, MAPK14, FOS, AKT1, STAT3, MAPK8
Toll-like receptor signaling pathway	18	STAT1, CD40, NFKBIA, PIK3CG, MAP2K1, MAPK3, NFKB1, IL6, JUN, PIK3CD, PIK3CA, RELA, MAPK1, MAPK14, FOS, IL8, AKT1, MAPK8
HIF-1 signaling pathway	13	PIK3CD, PIK3CA, MAPK1, RELA, PIK3CG, MAPK3, MAP2K1, NFKB1, IL6, EGFR, STAT3, AKT1, BCL2
PI3K–Akt signaling pathway	19	PIK3CG, MAPK3, MAP2K1, NFKB1, IL6, CREB1, TP53, IL2, HSP90AA1, IL4, PIK3CD, JAK2, SYK, PIK3CA, RELA, MAPK1, EGFR, AKT1, BCL2
Chemokine signaling pathway	17	STAT1, SRC, CCL2, NFKBIA, PIK3CG, MAP2K1, MAPK3, NFKB1, CXCL12, PIK3CD, JAK2, PIK3CA, RELA, MAPK1, IL8, AKT1, STAT3
Neurotrophin signaling pathway	16	NFKBIA, PIK3CG, MAPK3, MAP2K1, NFKB1, TP53, JUN, PIK3CD, CALM2, PIK3CA, RELA, MAPK1, MAPK14, AKT1, MAPK8, BCL2

### Experimental Investigations

#### Characterization of the HT Formula Chemical Constituents

**Figure 3:**
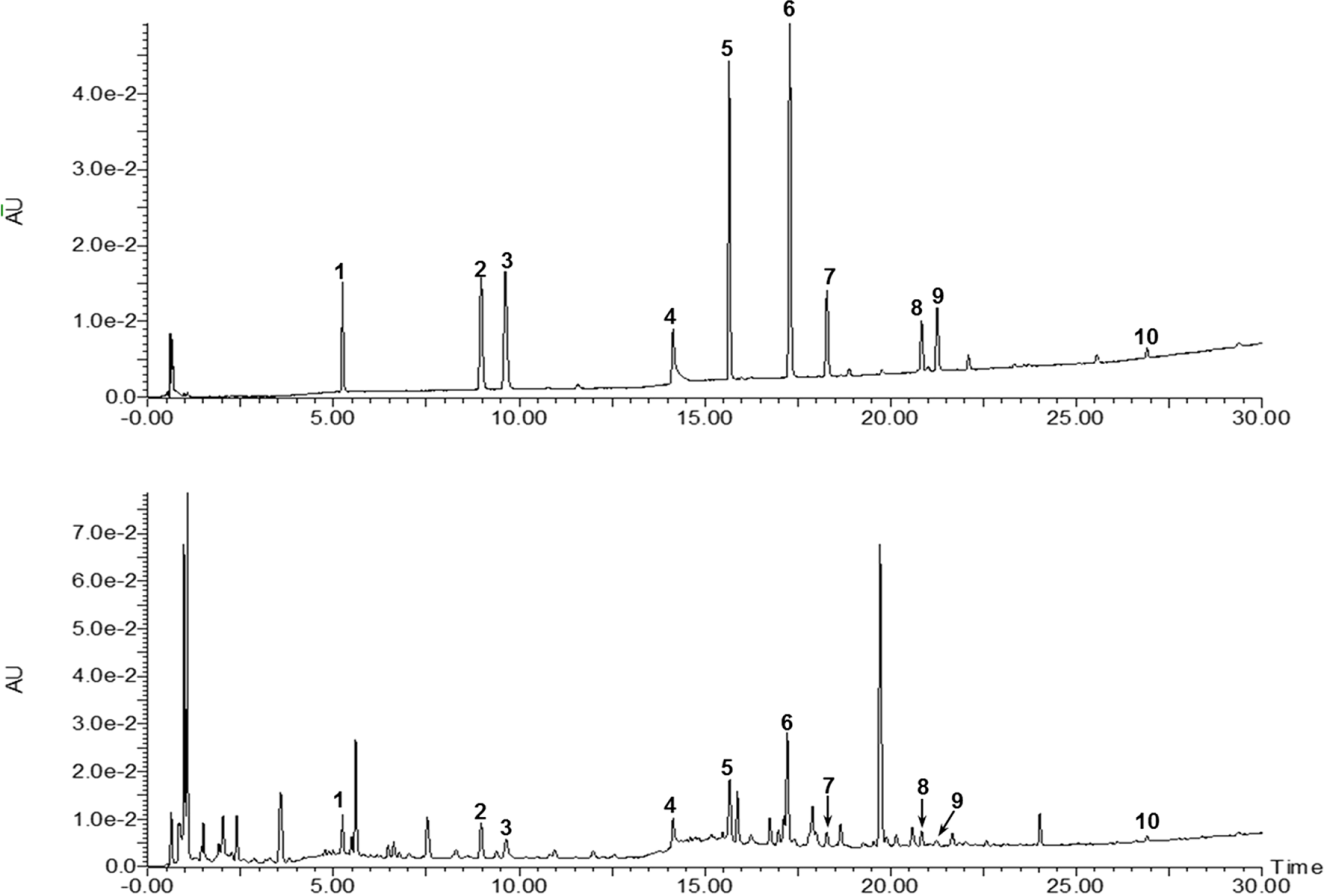
Ultraperformance liquid chromatography-photodiode array (UPLC-PDA) chromatograms of the mixed standards and HT. **1**: Danshensu, **2**: 3-caffeoylquinic acid, **3**: caffeic acid, **4**: paeoniflorin, **5**: rutin, **6**: quecertrin, **7**: rosmarinic acid, **8**: salvigenin, **9**: calycosin, and **10**: glyrrhizic acid.

A total of 10 compounds contained in the extract of the HT formula were quantitatively determined using the UPLC-photodiode array (UPLC-PDA) method. As illustrated in **Figure 3**, all of the compounds detected could be chromatographically separated without interferences. Moreover, the contents of the 10 compounds in the HT extract were quantified to be 0.143 ± 0.001% (danshensu), 0.083 ± 0.001% (3-caffeoylquinic acid), 0.061 ± 0.001% (caffeic acid), 0.611 ± 0.011% (paeoniflorin), 0.232 ± 0.001% (rutin), 0.202 ± 0.001% (quercetin), 0.052 ± 0.001% (rosmarinic acid), 0.104 ± 0.002% (salvigenin), 0.009 ± 0.001% (calycosin), and 0.131 ± 0.004% (glycyrrhizic acid), respectively.

#### Anti-Inflammatory Properties

**Figure 4:**
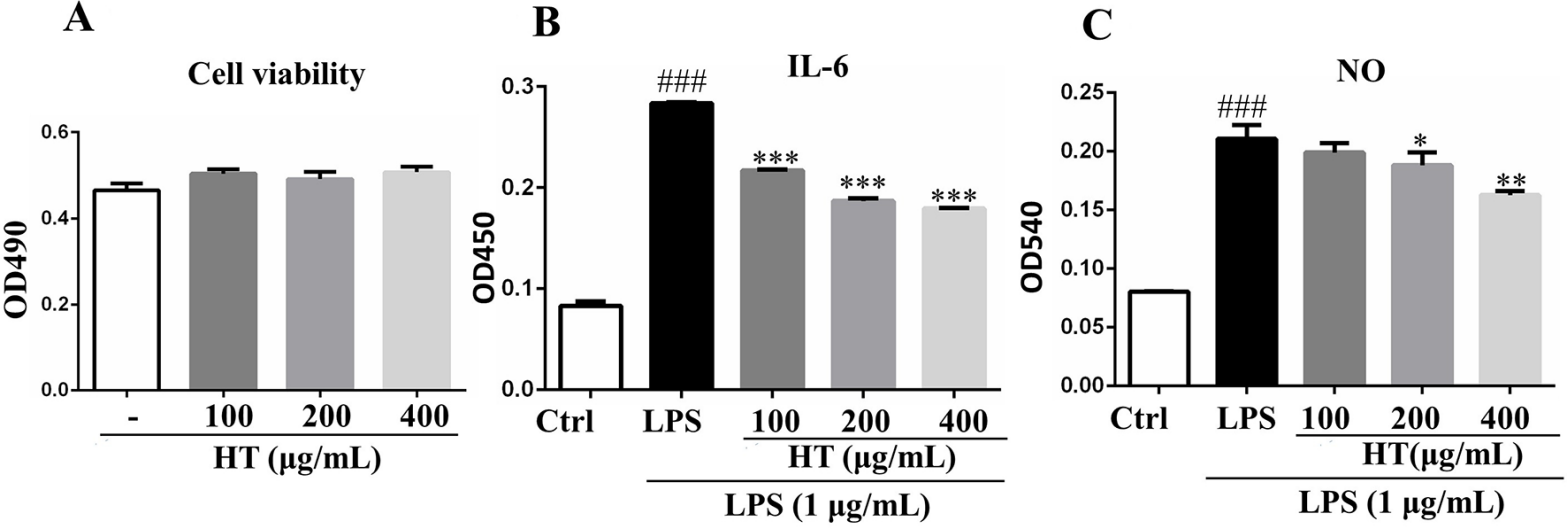
Effects of HT on RAW264.7 cells in the presence or absence of lipopolysaccharide (LPS). The cells were cotreated with the extracts in 96-well plate at indicated concentrations for 24 h; then, the cell viability was detected by 3-(4,5-dimethylthiazol-2-yl)-2,5-diphenyltetrazolium bromide (MTT) assay **(A)**. The cells were pretreated with the extracts at the concentrations of 100, 200, and 400 μg/ml in 24-well plate for 1 h, then stimulated with LPS (1 μg/ml) for 24 h; the supernatants were collected to determine cytokines secretion with ELISA kit **(B)** and NO release with Griess reagent **(C)**. Data are presented as the mean ± SD (*n* = 3). ^###^*P* < 0.001 vs. the Ctrl group. *P < 0.05, **P < 0.01, ***P < 0.001 vs. the LPS group.

**Figure 5:**
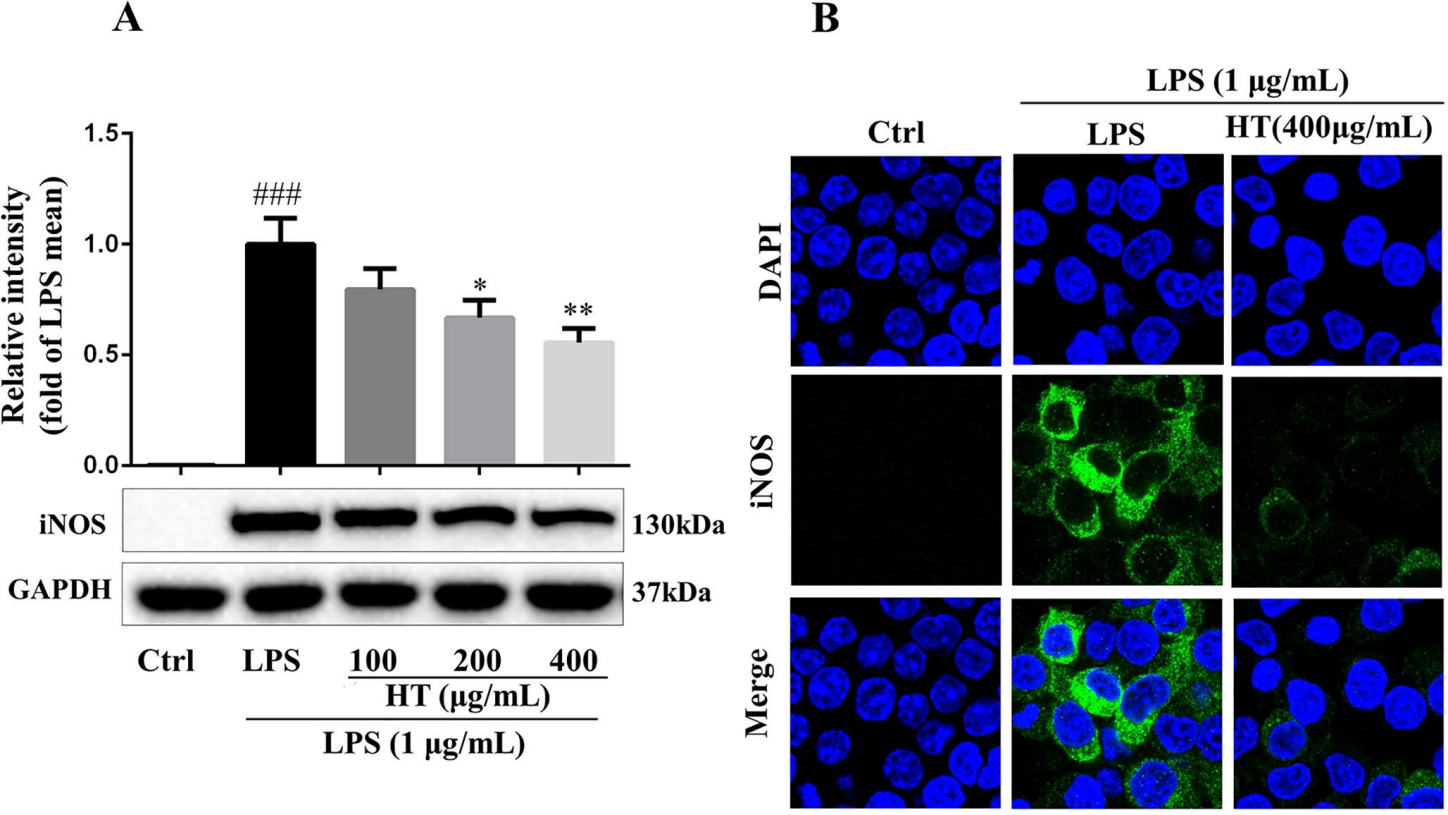
HT reduced inducible nitric oxide synthase (iNOS) expression in LPS-induced RAW264.7 cells. The cells were pretreated with HT extract at indicated concentrations in six-well plate for 1 h before stimulation with LPS (1 μg/ml) for 12 h; then, the expression of iNOS was examined by western blotting **(A)** and immunofluorescence staining **(B)**. Data are presented as the mean ± SD (*n* = 3). ^###^*P* < 0.001 vs. the Ctrl group. *P < 0.05, **P < 0.01 vs. the LPS group.

**Figure 6:**
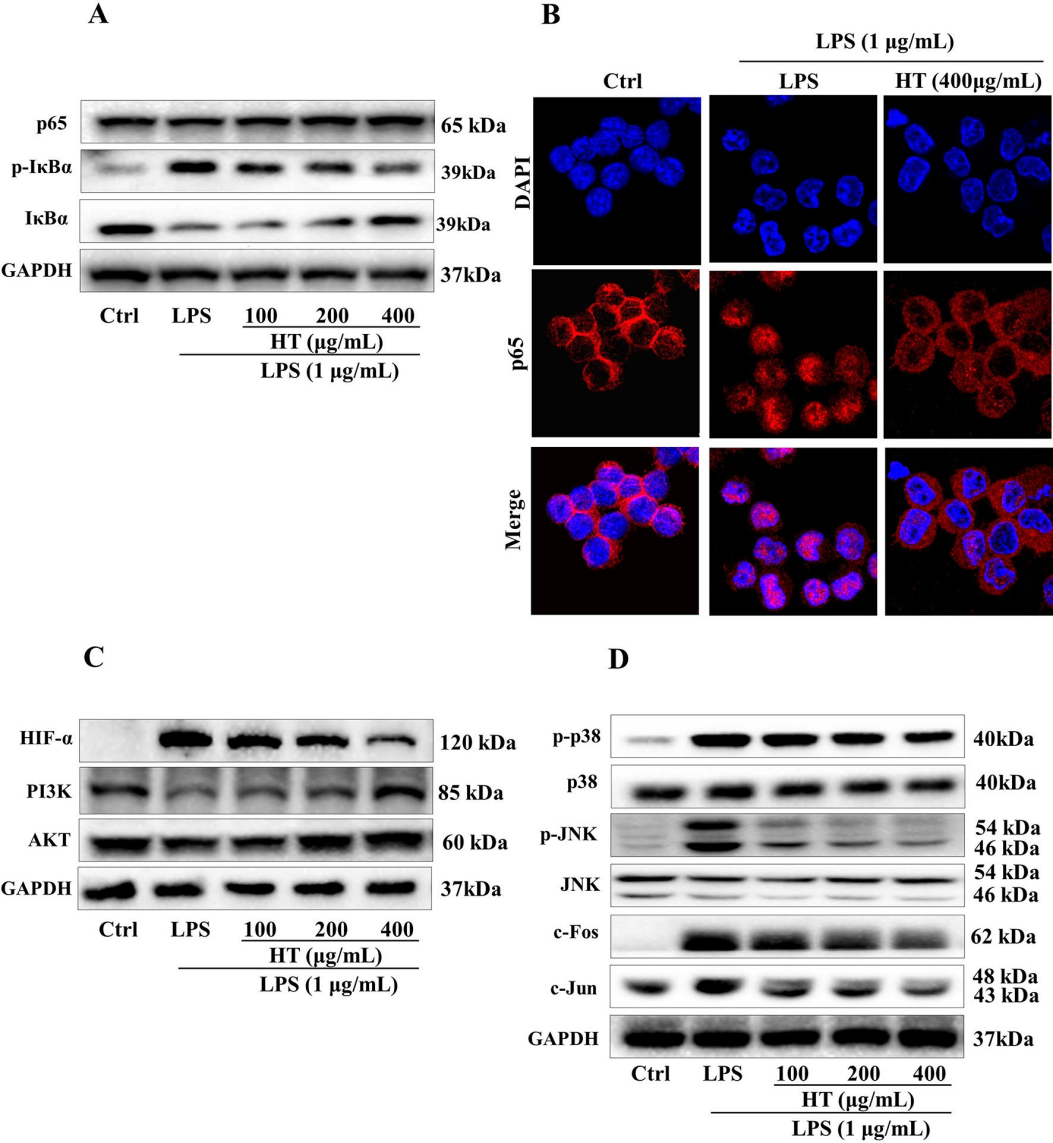
Effects of the HT extract on the relevant proteins in the LPS-induced RAW264.7 cells. Cells were pre-treated with the extract at the concentrations of 100, 200, 400 μg/mL in the 6-well plates for 1 h prior to the stimulation with LPS (1 μg/mL) for 12 h or 1 h, and the relevant proteins deduced from the computational predictions were analyzed by western blotting **(A**, **C** and **D)** and immunofluorescence staining **(B)** subsequently.

Results from MTT assay showed that the HT extract (≦400 μg/ml) was noncytotoxic to the RAW264.7 macrophages (**Figure 4A**). Under the safety dosages, the HT extract inhibited the interleukin 6 (IL-6) secretion (**Figure 4B**) and the NO production (**Figure 4C**) in a dose-dependent manner. The HT extract also reduced the excessive expression of iNOS (**Figure 5**). The HT extract blocked the activation of NF-κB (**Figures 6A, B**). In addition, the HT extract activated the phosphoinositide-3-kinase/AKT signaling pathway and inhibited the expression of hypoxia-inducible factor 1-alpha (**Figure 6C**). **Figure 6D** shows that the HT extract suppressed the expression of the mitogen-activated protein kinase (MAPK) family proteins including the p-p38 and p-JNK, and the HT extract also inhibited the activation of activator protein 1 (AP-1) through c-Fos and c-Jun.

## Discussion

RA is a chronic inflammatory systemic disease mainly affecting the joints, which is characterized by synovial hyperplasia and inflammatory cells infiltration, leading to the tissue destruction and functional disability (Scott and Wolfe F, 2010). RA treatment in TCM has a long history in China, and there have been several herbal formulae created by the famous TCM doctors based on their clinical experiences as well as the TCM theories considered effective, and the HT formula has been regarded as one of such. In this study, we aim to provide a comprehensive analysis of the HT formula integrating computational predictions and *in vitro* experimental methods, contributing to the quality control, product development, as well as the clinical applications of the formula.

According to the results from the pathway enrichment analysis, the major hubs of the HT formula were frequently involved with the chemokine signaling pathway, prolactin signaling pathway, T cell receptor signaling pathway, tumor necrosis factor (TNF) signaling pathway, Toll-like receptor signaling pathway, as well as HIF-1 signaling pathway. These interactions were associated with the pathological mechanisms of RA. Chemokine signaling pathway had been found to be a contributor for chronic inflammation among RA patients (Zhang et al., 2015). Prolactin signaling pathway perplexed with the progression of RA in a way through the incorporation between prolactin and other proinflammatory stimuli to activate macrophages (Tang et al., 2016). T cell receptor signaling pathway had been detected to participate in the initiation and progression of RA (Lundy et al., 2007). TNF signaling pathway was regarded as being related to inflammation, and the inhibitors targeting TNF receptors had been used to treat systemic inflammatory disorders since the 1990s (Scheinecker et al., 2012). Toll-like receptors were key mediators of inflammation in RA, and its signaling pathway was considered linked to the progression of the disease (Monaco et al., 2011). Studies had found out that HIF-1 signaling pathway could interact with Toll-like receptor signaling pathway to induce inflammation in RA (Hu et al., 2014). As deduced from the pathway enrichment results, HT formula mainly regulated the bone metabolism and inflammatory pathways when treating RA, indicating that the formula may play an important role in regulating the bone density among RA patients, as well as the progression of RA. There were 20 major hubs identified as being involved with the osteoclast differentiation. In addition, 19 major hubs considered belonging to the phosphatidylinositol 3′-kinase (PI3K)–Akt signaling pathway, and the PI3K–Akt signaling pathway had been considered important in regulating the differentiation and the formation of the osteoblasts (Xi et al., 2015). Besides, 16 major hubs were determined as belonging to the neurotrophin signaling pathway, which had been linked to osteogenesis (Su et al., 2016). Bone erosion had been regarded as one of the main features among patients suffering from chronic RA (Schett and Gravallese, 2012), and the results from the enrichment analysis of the major hubs of the HT formula shed light on its potential MOA in combating RA by regulating the biological pathways related to bone metabolism.

The computational predictions showed us that the HT formula could ameliorate RA through several signaling pathways by targeting different genes. Thus, we evaluated the results from the prediction with *in vitro* investigations. As illustrated in **Figure 3**, the 11 compounds contained in the HT formula were quantitatively determined using the UPLC-PDA methods, and these data provided us with the guidance for the quality control of the HT formula. As shown in **Figure 4**, the HT extract significantly reduced the secretion of IL-6 and NO under the nontoxic dosages, which indicated that the HT formula would be a potential anti-inflammatory formula in accordance with the prediction results. Furthermore, our experimental results suggested that the HT extract could decrease the LPS-induced excessive expression of the iNOS (**Figure 5**), which has been reported to promote the production of IL-6 and NO during the development of RA (Li et al., 2017; Liu et al., 2018). As well known, iNOS is the downstream signal in the network of inflammatory cascade reaction, and several signaling pathways including the Toll-like receptor signaling pathway (Li et al., 2019), PI3K–Akt signaling pathway (Cao et al., 2019), HIF-1 signaling pathway (Lee et al., 2018), and TNF signaling pathway (Pooladanda et al., 2019) from our prediction results have been reported to regulate inflammation by mediating the expression of iNOS. To further examine the underlying mechanism of the HT formula in regulating the signaling pathways and potentially ameliorating inflammation, it was first observed that the HT extract could block the activation of NF-κB (**Figures 6A, B**), which was the downstream of TLR4. As **Figure 6C** shows, the HT extract also activated the PI3K/AKT signaling pathway and inhibited the hypoxia-inducible factor 1-alpha expression. Besides, **Figure 6D** shows that the HT extract suppressed the MAPK family by interfering with p-p38 and p-JNK (**Figure 6D**), which was in accordance with our predication results as shown in **Table 2**. JNK has been reported to mediate the expression of AP-1, which was involved in the progression of RA (Hannemann et al., 2017; Yang et al., 2017; Kim et al., 2018). As is shown in **Figure 6D**, the HT extract could inhibit the activation of AP-1 composed with c-Fos and c-Jun.

In summary, we made a systematic analysis for the HT formula using computational prediction as well as *in vitro* investigational methods. Our experimental results were in good accordance with the results predicted from the network analysis of the HT formula, especially the anti-inflammatory properties of the formula. This study not only evaluated the anti-inflammatory effects of the HT formula when treating RA but also proposed the potentiality of the HT formula on regulating the bone metabolism in the body, which deserves future examination.

## Data Availability

All datasets generated for this study are included in the manuscript and the **Supplementary Files**.

## Author Contributions

ZW and K-GL designed and conducted the study with equal contribution. YH, GC, and HuY supervised the study. R-YH, HaY, S-HX, JL, and HZ provided the technical support and advices for the study. All authors contributed to the review and the approval of the final manuscript.

## Funding

We appreciate the financial support received from the National Natural Science Foundation of China (NSFC, No. 81470170), Macau Science and Technology Development Fund (013/2015/A1), University of Macau (MYRG2016-00144-ICMS-QRCM, MYRG2017-00178-ICMS, MYRG2018-00043-ICMS), Guangdong-Macao Traditional Chinese Medicine Technology Industrial Park (D-Pro-0274-2017), Natural Science Foundation of China (No.81774218), Guangdong Provincial Hospital of Chinese Medicine (No. YN2018ML08, YN2018ZD06), the Science and Technology Project of Guangdong Province (No. 2016A020226041), the 1010 Project of Guangdong Provincial Hospital of Chinese Medicine (No. YN10101906), Guangzhou Municipal Science and Technology Innovation Committee (No. 201710010076), and Science and Technology Planning Project of Guangdong Province (No. 2017B030314166) for this research.

## Conflict of Interest Statement

The authors declare that the research was conducted in the absence of any commercial or financial relationships that could be construed as a potential conflict of interest.
